# Antioxidant Activity of *Artocarpus heterophyllus* Lam. (Jack Fruit) Leaf Extracts: Remarkable Attenuations of Hyperglycemia and Hyperlipidemia in Streptozotocin-Diabetic Rats

**DOI:** 10.1100/tsw.2011.71

**Published:** 2011-04-05

**Authors:** Haidy S. Omar, Hesham A. El-Beshbishy, Ziad Moussa, Kamilia F. Taha, Abdel Nasser B. Singab

**Affiliations:** ^1^ Phytochemistry Department, National Organization of Drug Control and Research, Cairo, Egypt; ^2^ Medical Laboratories Technology Department, Faculty of Applied Medical Sciences, Taibah University, Madinah, Saudi Arabia; ^3^ Biochemistry Department, Faculty of Pharmacy, Al-Azhar University, Cairo, Egypt; ^4^ Chemistry Department, Faculty of Science, Taibah University, Madinah, Saudi Arabia; ^5^ Pharmacognosy Department, Faculty of Pharmacy, Ain Shams University, Cairo, Egypt

**Keywords:** Jack fruit leaves, hyperglycemia, hyperlipidemia, antioxidant

## Abstract

The present study examines the antioxidative, hypoglycemic, and hypolipidemic activities of *Artocarpus heterophyllus* (jack fruit) leaf extracts (JFEs). The 70% ethanol (JFEE), n-butanol (JFBE), water (JFWE), chloroform (JFCE), and ethyl acetate (JFEAE) extracts were obtained. Both JFEE and JFBE markedly scavenge diphenylpicrylhydrazyl radical and chelate Fe^+2^*in vitro*. A compound was isolated from JFBE and identified using 1D and 2D ^1^H- and ^13^C-NMR. The administration of JFEE or JFBE to streptozotocin (STZ)-diabetic rats significantly reduced fasting blood glucose (FBG) from 200 to 56 and 79 mg%, respectively; elevated insulin from 10.8 to 19.5 and 15.1 μU/ml, respectively; decreased lipid peroxides from 7.3 to 5.4 and 5.91 nmol/ml, respectively; decreased %glycosylated hemoglobin A1C (%HbA1C) from 6.8 to 4.5 and 5.0%, respectively; and increased total protein content from 2.5 to 6.3 and 5.7 mg%, respectively. Triglycerides (TG), total cholesterol (TC), low-density lipoprotein cholesterol (LDL-C), VLDL-C, and LDL/HDL ratio significantly declined by -37, -19, -23, -37, and -39%, respectively, in the case of JFEE; and by -31, -14, -17, -31, and -25%, respectively, in the case of JFBE; as compared to diabetic rats. HDL-C increased by +37% (JFEE) and by +11% (JFBE). Both JFEE and JFBE have shown appreciable results in decreasing FBG, lipid peroxides, %HbA1C, TC, LDL-C, and TG levels, and increasing insulin, HDL-C, and protein content. The spectrometric analysis confirmed that the flavonoid isolated from JFBE was isoquercitrin. We can conclude from this study that JFEE and JFBE exert hypoglycemic and hypolipidemic effects in STZ-diabetic rats through an antioxidative pathway that might be referred to their flavonoid contents.

### INTRODUCTION

Diabetes mellitus is a chronic metabolic disease that affects 5% of the world population[1]. It is caused by an inherited or acquired deficiency of insulin secretion that results in an increased blood glucose level, which in turn produces adverse effects on different body systems[2,3,4]. There are limitations to currently available drugs, which merit the consideration of new agents with the potential for greater efficacy or fewer side effects[5].

It is estimated that different species of plants are used as folk medicines to treat diabetes[6]. Among these is the jack fruit (*Artocarpus heterophyllus* Lam., family Moraceae), an exotic fruit grown in tropical climates. The fruit has an average weight of 10 kg. The yellowish bulbs constituting the perianth portion of the fruit are fleshy, fibrous, and rich in sugars as well as carotenoids. It is considered a rich source of carbohydrates, minerals, carboxylic acids, dietary fiber, and vitamins such as ascorbic acid and thiamine[7]. Different classes of flavonoids are abundant in the jack fruit plant[8,9]. The various parts of the jack fruit have been used in traditional medicines[10]. In the market, a few jack fruit products, such as jack fruit with honey, canned jack fruit, and jack fruit flavors, are available. Jack fruit powders are used in instant soups, snacks, bakery products, beverages, dairy products, candy, ice cream, baby foods, pasta, etc.[11]. Several reports have cited the antidiabetic effects of jack fruit extracts (JFEs), which could be attributed to its high proanthocyanidin and flavonoid contents[12,13,14], through inhibition of lipid peroxide formation[15], and *via* an $\alpha$-amylase inhibitory effect, indicating that it could act as a starch blocker to decrease postprandial glucose level[16]. In addition, it was reported that JFEs possess anti-inflammatory and antibacterial activity[9,17].

This study was undertaken to investigate the *in vitro* antioxidant potentials of JFEs through measuring diphenylpicrylhydrazyl ($DPPH^{\cdot}$) radical scavenging and ferrous ion ($Fe^{++}$) chelating activities. Also, the study endeavored to investigate *in vivo* the hypoglycemic and hypolipidemic effects of selected JFEs in streptozotocin (STZ)-induced hyperglycemia in rats and to gain insight into the possible mechanisms. Finally, we wished to isolate the active compound from the most powerful antioxidant, hypoglycemic, and hypolipidemic JFEs.

### MATERIALS AND METHODS

#### Chemicals

STZ and glibenclamide (GLB) were purchased from Sigma-Aldrich (U.S.A.). All other chemicals were purchased from Merck (Germany) and Acros Chemicals (Belgium).

#### Plant Material

The leaves of *A. heterophyllus* Lam. (jack fruit) were collected during 2007–2008 from a botanical garden in Aswan, Egypt. The plants were kindly authenticated and coded by the Orman Garden Herbarium, Cairo, Egypt.

#### Preparation of Plant Extracts

The air-dried, powdered jack fruit leaves were subjected to preliminary phytochemical screening. The results revealed the presence of carbohydrates, flavanoids, glycosides, sterols, and tannins; however, steam volatiles and saponins were absent.

The air-dried jack fruit leaves (2 kg) were finely powdered and macerated in 3.5 L 70% ethanol (1:0.75 w/v) until exhaustion, followed by filtration to yield the ethanol crude extract (JFEE), which was evaporated until dryness under reduced pressure to obtain a dark-green viscous mass with a yield of 9.85% w/w (197 g) after lyophilization. The extract was suspended in dist. $H_2O$ and successively fractionated using different solvents to obtain different extracts; namely, petroleum ether (JFPE) yield (23% w/w, 45.5 g), chloroform (JFCE) yield (5% w/w, 11.5 g), ethyl acetate (JFEAE) yield (3.5% w/w, 7 g), n-butanol (JFBE) yield (10.6% w/w, 21 g), and water fraction (JFWE) yield (22.8% w/w, 45 g).

### Spectrometric Analysis

Jack fruit leaves (750 g) were extracted repeatedly with 70% ethanol at room temperature. The ethanolic crude extract (JFEE) was concentrated under vacuum to yield a residue of 74 g (9.8% w/w). The extract was suspended in water and partitioned with petroleum ether, chloroform, ethyl acetate, and n-butanol. The JFBE (20 g, 27.02% w/w) was applied to Di-anion column (Ø 1 m × 5 cm, 200 g) and eluted with 500 mL methanol:water (50:50) to give three fractions. The third fraction yielded 35 subfractions. Fraction numbers 30–35 (0.12 g, 6% w/w) were applied on silica gel column (110 g, Ø 55 × 11 cm) using ethyl acetate:formic acid:acetic acid:water 100:11:11:2, 100:11:11:4, 100:11:11:6, 100:11:11:8, and 100:11:11:10 to give five fractions that were pooled together, concentrated under reduced pressure, and purified on a Sephadex LH-20 column using 90% methanol as eluent, followed by crystallization, to give 40 mg of a yellow powder that elicited a brown color at 254 nm and produced a bright yellow color upon spraying with $AlCl_3$, which was identified spectroscopically using both 1D and 2D $^1$H- and $^{13}$C-NMR techniques. The $^1$H- and $^{13}$C-NMR spectra of this compound were recorded at Taibah University, Saudi Arabia, on an Avance II Bruker FT-NMR spectrometer 400 (400 MHz) using $CD_3OD$ or DMSO-d6 as solvents and TMS as an internal standard. Chemical shifts are expressed as $\delta$ ppm units.

### *In Vitro* Antioxidant Activity of Various JFEs

#### *DPPH$^{\cdot}$ Assay*

The hydrogen atom or electron donation ability of the JFEs was measured as a result of the bleaching of purple-colored methanol solution of DPPH$^{\cdot}$. Briefly, each of the JFEs (JFEE, JFBE, JFWE, JFCE, and JFEAE) were used in this experiment at concentrations of 0.2, 0.4, and 0.6 mg/mL for each of the mentioned extracts in triplicate; 50 µL of each concentration was added to 5 mL of 0.004% methanol solution of DPPH$^{\cdot}$. After a 30-min incubation at room temperature, the absorbance was recorded against blank at 517 nm[18]. Vitamin E was used as a reference antioxidant. The percentage of inhibition of DPPH$^{\cdot}$ free radical scavenging activity = $[A_c – A_s/A_c]$□□ × 100, where, $A_c$: absorbance of control and $A_s$: absorbance of sample.

#### *Ferrous Ion ($Fe^{++}$) Chelating Activity*

The $Fe^{++}$ chelating ability of JFEs was monitored by the absorbance of $Fe^{++}$-ferrozine complex at 562 nm. Briefly, JFEE, JFBE, JFWE, JFCE, and JFEAE were used at concentrations of 0.2, 0.4, and 0.6 mg/mL in triplicate. A volume of 0.4 mL of each extract was added to 0.2 mL 2 mM $FeCl_2$. The reaction was initiated by addition of 0.4 mL 5 mM ferrozine. The total volume was adjusted to 4 mL with ethanol. Then, the mixture was shaken vigorously and left at room temperature for 10 min, followed by measuring absorbance at 562 nm[19]. Absorbance of the solution was then measured spectrophotometrically at 562 nm[19]. Vitamin E was used as a reference antioxidant. The percentage of inhibition of $Fe^{++}$-ferrozine complex formation was calculated by using the equation: $Fe^{++}$ chelating effect percentage = $[1 – A_s/A_c] \times 100$, where $A_C$: absorbance of control and $A_S$: absorbance of sample.

### Experimental Animals

Male Wistar rats weighing 170–250 g were used in this study. Animals were maintained under standard conditions of temperature (24 ± 5°C) and relative humidity (55 ± 5%), with a regular 12 h light:12 h dark cycle, and allowed free access to standard laboratory food and water 7 days before starting the experiment and during the whole period of the experiment. All animals were fed with common pellet diets and water *ad libitum*. All animals were treated humanely in accordance with the guideline for care of animals as set by the World Health Organization.

### Induction of Diabetes Mellitus

Diabetes was induced in the rats by a single intraperitoneal (i.p.) injection of freshly prepared STZ (60 mg/kg b.w.) in normal saline. Two days after STZ administration, blood samples were obtained from the tips of the rat's tail and the fasting blood glucose (FBG) levels determined using a OneTouch® Ultra® glucometer (LifeScan, U.S.A.) to confirm diabetes. The diabetic rats exhibiting blood glucose levels above 190 mg% were included in this study[20]. The biochemical effects of JFEE and JFBE were compared to GLB (a reference hypoglycemic drug).

### Experimental Design and Treatment Regimen

Rats were subdivided randomly into five groups (eight rats/group) and treated as follows:-

- Group I: Normal control rats received 0.1 mL DMSO and 0.5 mL 5% Tween 80 for 10 days by oral lavage.
- Group II: STZ-diabetic rats received 0.1 mL DMSO and 0.5 mL 5% Tween 80 for 10 days by oral lavage.
- Group III: Diabetic rats orally received GLB (600 μg $kg^{-1}$ $day^{-1}$) in 0.1 mL DMSO and 0.5 mL 5% Tween 80 for 10 days by oral lavage[21].
- Group IV: STZ-diabetic rats orally received JFEE dissolved in 0.1 mL DMSO and 0.5 mL 5% Tween 80 at a dose of 200 mg $kg^{-1}$ $day^{-1}$ for 10 days by oral lavage[22].
- Group V: STZ-diabetic rats orally received JFBE dissolved in 0.1 mL DMSO and 0.5 mL 5% Tween 80 at a dose of 200 mg $kg^{-1}$ $day^{-1}$ for 10 days by oral lavage[22].

On the 11th day, the fasting rats were subjected to light ether anesthesia and killed by cervical dislocation. Trunk blood was collected into heparinized chilled tubes containing sodium fluoride (to inhibit glycolysis). Serum was separated by centrifugation at 4°C and stored at -20°C until further use.

### Measurements of Biochemical Parameters

Sera were assayed for FBG, total protein content, triglycerides (TG), total cholesterol (TC), high-density lipoprotein cholesterol (HDL-C), and low-density lipoprotein cholesterol (LDL-C) using kits purchased from Spinreact, Spain. The very-low-density lipoprotein cholesterol (VLDL-C) was calculated by dividing the values of TG by 5[23]. The LDL/HDL ratio was also calculated. The lipid peroxides (expressed as thiobarbituric acid reactive substance, TBARS) was measured in serum[24]. Serum insulin level was estimated using an enzyme immunoassay kit purchased from SPI-BIO (Society of Pharmacology and Immunology-BIO), France. The whole blood with EDTA was used to estimate glycosylated hemoglobin percentage (%HbA1C) using a diagnostic kit purchased from Beckman Coulter, U.K.

### Statistical Analysis

Data were expressed as means ± SEM. Statistical comparison between different groups were done using one-way analysis of variance (ANOVA), followed by the Tukey-Kramer multiple comparison test, to judge the difference between various groups. Significance was accepted at $p < 0.05$.

## RESULTS

### Structure Elucidation of a Compound Isolated from Jack Fruit Leaves

Partial identification of the chemical formula of the unknown compound isolated from JFBE was based on combined data gathered from elemental analysis and mass spectral measurement (LC/MS). The former technique yielded the weight percent of carbon and hydrogen, and revealed the absence of both nitrogen and sulfur (C, 54.09; H, 4.41; N, 0; S, 0), whereas the latter technique gave a nominal mass of 465.2 $[MH]^+$. The number of carbon and hydrogen atoms was 21 and 20, respectively. A partial molecular formula of $C_{21}H_{20}$ was subsequently obtained.

The DEPT-135 $^{13}$C-NMR revealed the presence of five aromatic CHs (δ 123.0, 117.8, 116.1, 100.0, and 94.8), five cyclic methine (CH) protons (δ 105.4, 77.2, 75.1, 73.2, and 70.1), and one methylene ($CH_2$) group (δ 62.0). The $^{13}$C-NMR indicated the presence of 10 quaternary carbon atoms in addition to the remaining signals observed in the DEPT spectrum. The signals for the quaternary carbons stem from one carbonyl group (δ 179.5) and nine aromatic carbons (δ 166.3, 163.0, 158.8, 158.5, 150.0, 145.8, 135.8, 122.9, and 105.6). Interestingly, the total number of carbons observed in the $^{13}$C-NMR was 21, indicating that the structure contains no symmetry. The number of protons obtained from the DEPT spectrum summed to 12 protons (Table 1). The remaining eight protons were attached to oxygen atoms. This suggested the presence of at least nine oxygen atoms in the molecular structure, as we have eight hydroxyl groups and a carbonyl group. Indeed, we incorporated nine oxygen atoms into the partial molecular formula that was derived earlier to get a molecular formula of $C_{21}H_{20}O_9$ (mass of 416). The missing mass of 48 corresponds to three oxygen atoms, suggesting that the structure possesses three nonprotonated oxygen atoms, part of a ring system or connecting rings together. In accordance with this analysis, a molecular formula of $C_{21}H_{20}O_{12}$ (mass of 464) was derived. The $^1$H-NMR revealed the presence of 12 protons, which corroborated DEPT data. The remaining eight hydroxyl protons exchanged rapidly and therefore exhibited no absorption signals. The five aromatic protons were assigned to two aromatic rings [δ 6.40 (d, $J$ = 2.4 Hz, 1H), 6.20 (d, $J$ = 2.0 Hz, 1H)] and were correlated to each other *via* COSY. The small $J$ value of the coupling constant indicated long-range coupling and the $^{13}$C chemical shifts that were correlated *via* HSQC analysis (δ 94.8 and 100.0, respectively) suggested that the upfield absorption of carbon signals stems from the presence of a hydroxyl group separating the two aromatic methines. The three remaining aromatic methine groups were present on the second aromatic ring [δ 7.84 (d, $J$ = 2.0 Hz, 1H), 7.59 (dd, $J$ = 8.4, 2.0 Hz, 1H), 6.86 (d, $J$ = 8.4 Hz, 1H)] and correlated by COSY, which revealed vicinal coupling ($^3J$ = 8.4 Hz) between the second and third signal, and long-range coupling ($^4J$ = 2.0 Hz) between the first and second signal. This coupling pattern indicated two separated spin systems in which the two strongly coupled vicinal protons were separated from the third one by a substituent.

The $^{13}$C chemical shifts that were correlated *via* HSQC analysis (δ 116.1, 123.0, and 117.8, respectively) suggested that the upfield absorption of the first and third carbon stems from the presence of two adjacent hydroxyl groups in the 3 and 4 aromatic positions, which separated the two spin systems. It is noted that all these protons have been correlated by HSQC to six carbons, which were connected to oxygen atoms as their carbon chemical shifts range from δ 62.0 to 105.4. The most characteristic proton appeared as doublet at δ 5.16 (d, $J$ = 7.9 Hz) and was correlated to the carbon absorption at δ 105.4. This corresponds to the anomeric carbon of the glycosidic bond. The remaining alkyl protons absorbed from δ 3.87 to 3.44 and have been attributed to the cyclic protons of glucose and its alkyl $CH_2$ group (Fig. 1). According to the analysis presented, we propose that the unknown is 2-(3,4-dihydroxyphenyl) -3- (β-D-

**TABLE 1**
**$^{1}H$- and $^{13}C$-NMR Data of Isoquercitrin**

| $^{1}H$-NMR | Hydrogen Number | Isoquercitrin Protons |
|---|---|---|
| | 2` | 7.84 (1H, d, J = 2.0 Hz) |
| | 6` | 7.59 (1H, dd, J = 8.4, 2.0 Hz) |
| | 5` | 6.86 (1H, d, J = 8.4 Hz) |
| | 8 | 6.40 (1H, d, J = 2.4 Hz) |
| | 6 | 6.20 (1H, d, J = 2.0 Hz) |
| | 1`` | 5.16 (1H, d, J = 7.9 Hz) |
| | 2``- 6`` | 3.87- 3.44 (6H) |
| **$^{13}C$-NMR** | **Carbon Number** | **Isoquercitrin Carbons** |
| | 2, 3, 4, 5, 6, 7, 8, 9, 10 | 158.8, 135.8, 179.5, 163.0, 100.0, 166.3, 94.8, 158.5, 105.6 |
| | 1', 2', 3', 4', 5', 6' | 122.9, 117.8, 145.8, 150.0, 116.1, 123.0 |
| | 1", 2", 3", 4", 5", 6" | 105.4, 73.2, 75.1, 70.0, 77.2, 62.0 |

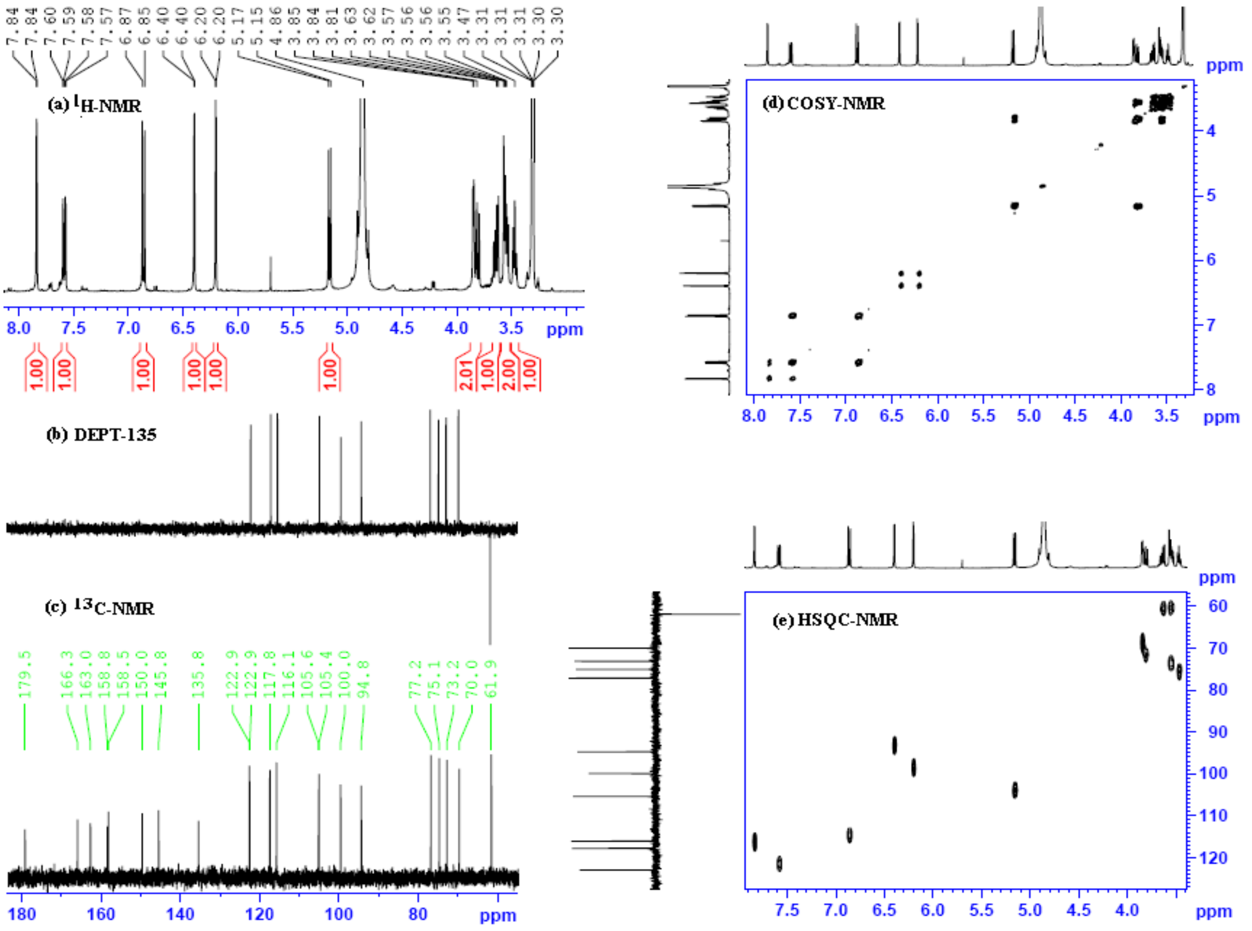

**FIGURE 1.** Spectra of the isolated compound from JFBE. (a) $^{1}H$-NMR ($CD_3OD$, 400 MHz); (b) DEPT-135 ($CD_3OD$, 100 MHz), CH (positive phase), $CH_2$ (negative phase); (c) $^{13}C$-NMR ($CD_3OD$, 100 MHz); (d) COSY-NMR; and (e) HSQC-NMR.

glucofuranosyloxy) -5,7-dihydroxy-4H-1-benzopyran-4-one, also named as quercetine-3-*O*-β-glucopyranoside or isoquercitrin (Fig. 2). The stereochemistry of the glycosidic bond of the anomeric carbon has been assigned as β based on the coupling constant of 1" CH ($J$ = 7.9 Hz) since the α-anomer exhibited a smaller coupling constant for the 1" CH ($J$ = 1.0 Hz). The $^{1}$H- and $^{13}$C-NMR data of isoquercitrin are presented in Table 1 and are as follows: $^{1}$H-NMR ($CD_3OD$, 400 MHz) δ 7.84 (d, $J$ = 2.0 Hz, 1H, Ar-H), 7.59 (dd, $J$ = 8.4, 2.0 Hz, 1H, Ar-H), 6.86 (d, $J$ = 8.4 Hz, 1H, Ar-H), 6.40 (d, $J$ = 2.4 Hz, 1H, Ar-H), 6.20 (d, $J$ = 2.0 Hz, 1H, Ar-H), 5.16 (d, $J$ = 7.9 Hz, 1H), 3.87–3.78 (m, 2H), 3.67–3.61 (m, 1H), 3.58–3.52 (m, 2H), 3.49–3.44 (m, 1H); $^{13}$C-NMR ($CD_3OD$, 100 MHz) δ 179.5 (C=O), 166.3 (ArC), 163.0 (ArC), 158.8 (ArC), 158.5 (ArC), 150.0 (ArC), 145.8 (ArC), 135.8 (ArC), 123.0 (ArCH), 122.9 (ArC), 117.8 (ArCH), 116.1 (ArCH), 105.6 (ArC), 105.4 (CH), 100.0 (ArCH), 94.8 (ArCH), 77.2 (CH), 75.1 (CH), 73.2 (CH), 70.1 (CH), 62.0 ($CH_2$). The structural assignment is consistent with that found in the literature as we further confirmed it by comparison of the $^{1}$H- and $^{13}$C-NMR results with those reported[25].

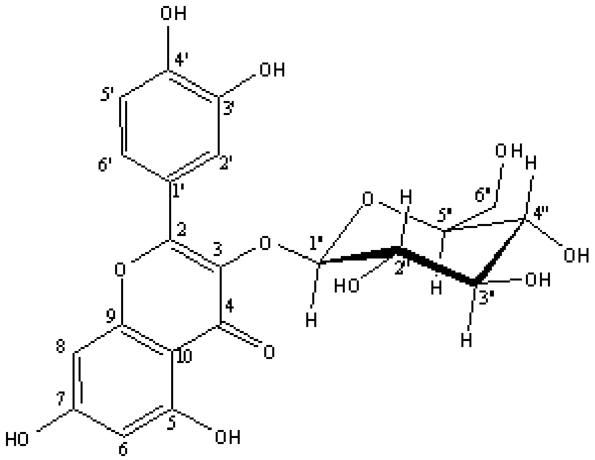

**FIGURE 2.** Isoquercitrin isolated from JFBE. Chemical formula: $C_{21}H_{20}O_{12}$; LC/MS: Analytical calculation for $C_{21}H_{20}O_{12}$: 465.2 $[MH]^{+}$. Found: 465.3; Elemental analysis: C, 54.09; H, 4.41.

### *In Vitro* Antioxidative Effect of JFEs

JFEE, JFBE, JFWE, JFCE, and JFEAE were subjected to *in vitro* antioxidant evaluation using two different methods: DPPH$^{\cdot}$ assay and $Fe^{++}$chelating activity. The results revealed that the best fraction that exhibited *in vitro* antioxidant activity was JFEE, followed by JFBE and, to a lesser extent, JFWE (Figs. 3 and 4). The scavenging rates of JFEE to DPPH$^{\cdot}$ were 21, 32, and 51% at concentrations of 0.2, 0.4, and 0.6 mg/mL, respectively. The percentages were 15, 25, and 33% in the case of JFBE at concentrations of 0.2, 0.4, and 0.6 mg/mL, respectively. The percentages of $Fe^{++}$ chelating activity of JFEE were 62, 75, and 78% at concentrations of 0.2, 0.4, and 0.6 mg/mL, respectively. The percentages were 65, 76, and 77% in the case of JFBE at concentrations of 0.2, 0.4, and 0.6 mg/mL, respectively. Vitamin E used in the same concentration as JFEs exhibited %$Fe^{++}$ chelating activity of 50, 68, and 68% and % DPPH$^{\cdot}$ scavenging activity of 15, 22, and 27%, respectively.

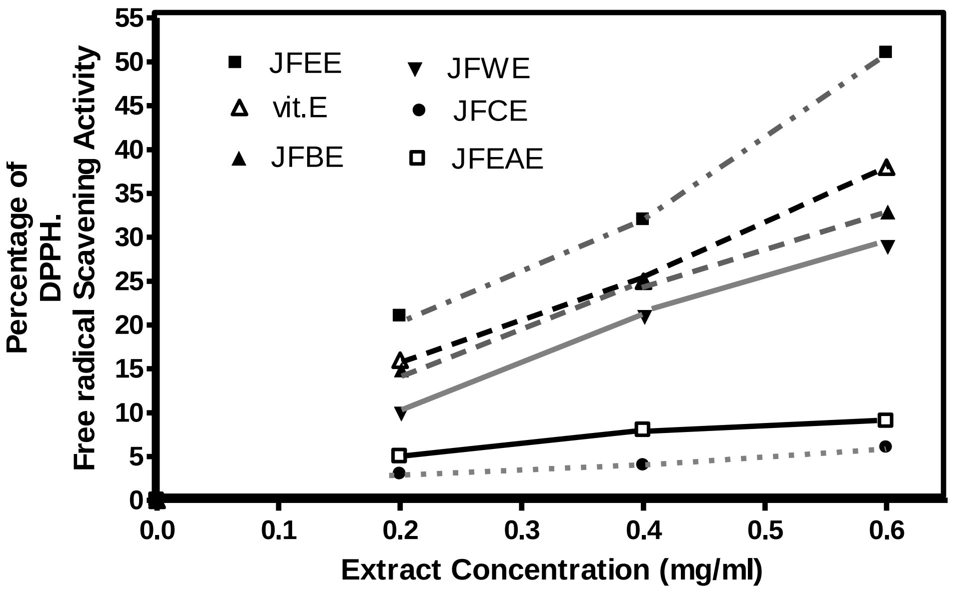

**FIGURE 3.** DPPH˙ free radical scavenging effect of different JFEs: JFEE, JFBE, JFWE, JFCE, and JFEAE in reference to vitamin E. Tests were carried out in triplicate.

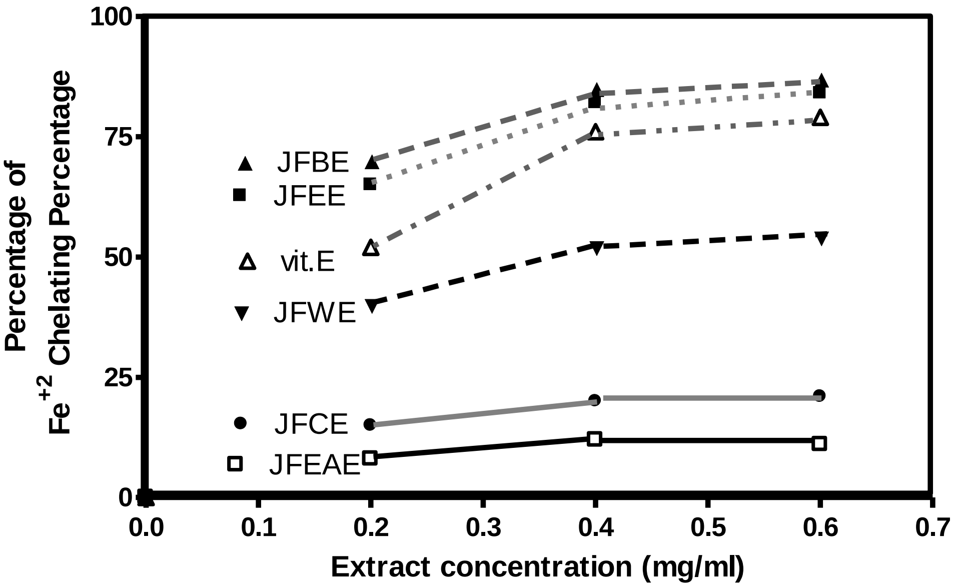

**FIGURE 4.** $Fe^{++}$ chelating effect of different JFEs: JFEE, JFBE, JFWE, JFCE, and JFEAE in reference to vitamin E. Tests were carried out in triplicate.

### Effect of JFEE and JFBE on Serum Proteins of Diabetic Rats

Injection of STZ into rats produced a significant decline in serum protein content by -64%, $p < 0.05$, as compared to normal rats (Table 2). Administration of GLB, JFEE, or JFBE to STZ-diabetic rats elicited a significant elevation in serum protein content by +104, +152, and +128%, respectively, $p < 0.05$, as compared to STZ-diabetic rats (Table 2).

**TABLE 2**
**Influence of Oral Intake of JFEE, JFBE (200 mg $kg^{-1}$ $day^{-1}$), or GLB (0.5 mg $kg^{-1}$ $day^{-1}$) for 10 Days to STZ-Diabetic Rats on Serum Protein Content, FBG, Lipid Peroxides Expressed as TBARS, %HbA1C, and Insulin**

| Group | Description | Protein (mg%) | FBG (mg%) | HbA1C (%) | Insulin (μU/mL) | TBARS (nmol/mL) |
|---|---|---|---|---|---|---|
| Group I | Normal control rats | 7.0 ± 0.2 | 55.0 ± 6.8 | 3.4 ± 0.05 | 21 ± 1.8 | 5.1 ± 0.06 |
| Group II | STZ-diabetic rats | 2.5 ± 0.2† | 200.0 ± 11.5† | 6.8 ± 0.1† | 10.8 ± 0.8† | 7.3 ± 0.07† |
| | % Change from group I | -64% | +264% | +100% | -49% | +43% |
| Group III | STZ-GLB–treated rats | 5.1 ± 0.3* | 142.0 ± 5.3* | 5.8 ± 0.1* | 13.6 ± 1.8 | 6.1 ± 0.04* |
| | % Change from group II | +104% | -29% | -15% | +26% | -16% |
| Group IV | STZ-JFEE–treated rats | 6.3 ± 0.2* | 56.0 ± 7.1* | 4.5 ± 0.1* | 19.5 ± 1.2* | 5.4 ± 0.035* |
| | % Change from group II | +152% | -72% | -34% | +81% | -26% |
| Group V | STZ-JFBE–treated rats | 5.7 ± 0.2* | 79.0 ± 8.1* | 5.0 ± 0.1* | 15.1 ± 1.3 | 5.9 ± 0.03* |
| | % Change from group II | +128% | -61% | -26% | +40% | -19% |

Values are expressed as means ± SEM (n = 8).

† Significant differences $p < 0.05$, as compared with normal control animals (group I).

* Significant differences $p < 0.05$, as compared with STZ-treated animals (group II).

### Hypoglycemic and Lipid Peroxide Inhibitory Effects of JFEE and JFBE

Injection of STZ into rats exhibited significant elevations in FBG, %HbA1C, and TBARS levels by +264, +100 and +43%, respectively, $p < 0.05$, as compared to normal control rats (Table 2). These changes were concomitant with a significant decline in serum insulin level by -49%, $p < 0.05$, as compared to normal rats. Administration of GLB to STZ-diabetic rats produced significant declines in FBG, %HbA1C, and TBARS levels by -29, -15, and -16%, respectively, $p < 0.05$, associated with a significant increase in serum insulin level by +26%, as compared to STZ-diabetic rats. Administration of JFEE to STZ-diabetic rats elicited a significant increase in serum insulin by +81%, joined with a significant decrease in FBG, %HbA1C, and TBARS levels by -72, -34, and -26%, respectively, $p < 0.05$, as compared to STZ-diabetic rats. On the other hand, administration of JFBE to STZ-diabetic rats elicited lesser changes than in the case of JFEE intake. These changes involved a significant increase in serum insulin by +40%, associated with a significant decrease in FBG, %HbA1C, and TBARS levels by -61, -26, and -19%, respectively, $p < 0.05$, as compared to STZ-diabetic rats (Table 2).

### Hypolipidemic Effects of JFEE and JFBE

Injection of STZ into rats produced a significant elevation in serum TC, LDL-C, VLDL-C, and TG levels by +37, +40, +69, and +69, respectively, $p < 0.05$, as compared to normal rats. These significant rises were accompanied by a significant decline of plasma HDL-C by -33%; $p < 0.001$, as compared to normal rats (Table 3). The atherogenic index LDL/HDL ratio was significantly increased in STZ-diabetic rats by +105%; $p < 0.05$, as compared to normal controls (Table 3). The oral supplementation of GLB to STZ-diabetic rats produced a significant decline in serum VLDL-C, TG, and LDL/HDL ratio by -21, -21, and -14%, $p < 0.05$, respectively, as compared to STZ-diabetic rats. The TC and LDL-C significantly declined by -12 and -8%, respectively; however, HDL-C increased significantly by 7%, $p < 0.05$, as compared to STZ-diabetic rats (Table 3). The oral intake of JFEE to STZ-diabetic rats showed a significant decline in serum TC, LDL-C, LDL/HDL ratio, VLDL-C, and TG by -19, -23, -39, -37, and -37%, respectively, $p <$

**TABLE 3**
**Influence of Oral Intake of JFEE, JFBE (200 mg $kg^{-1}day^{-1}$), or GLB (0.5 mg $kg^{-1}day^{-1}$) for 10 Days to STZ-Diabetic Rats on Serum Lipid Profiles Including TC, LDL-C, HDL-C, LDL/HDL ratio, VLDL-C, and TG**

| Group Description | | TC (mg%) | LDL-C (mg%) | HDL-C (mg%) | LDL/HDL Ratio | VLDL-C (mg%) | TG (mg%) |
|---|---|---|---|---|---|---|---|
| Group I | Normal control rats | 190 ± 3.7 | 86 ± 4.2 | 40 ± 1.6 | 2.15 ± 0.02 | 26 ± 1.6 | 130 ± 8.0 |
| Group II | STZ-diabetic rats | 260 ± 11† | 120 ± 2.6† | 27 ± 1.5† | 4.4 ± 0.03† | 44 ± 1.8† | 220 ± 9.0† |
| | % Change from group I | +37% | +40% | -33% | +105% | +69% | +69% |
| Group III | STZ-GLB–treated rats | 228 ± 9.6 | 110 ± 7.2 | 29 ± 1.1 | 3.8 ± 0.09* | 34.6 ± 1.4* | 173 ± 6.4* |
| | % Change from group II | -12% | -8% | +7% | -14% | -21% | -21% |
| Group IV | STZ-JFEE–treated rats | 210 ± 8.8* | 93 ± 6.9* | 37 ± 1.3* | 2.7 ± 0.03* | 27.8 ± 1.5* | 139 ± 5.8* |
| | % Change from group II | -19% | -23% | +37% | -39% | -37% | -37% |
| Group V | STZ-JFBE–treated rats | 223 ± 7.6* | 100 ± 3.0* | 30 ± 1.0* | 3.3 ± 0.06* | 30.2 ± 1.8* | 151 ± 7.5* |
| | % Change from group II | -14% | -17% | +11% | -25% | -31% | -31% |

Values are expressed as means ± SEM (n = 8).

† Significant differences $p < 0.05$, when compared with control animals (group I).

* Significant differences $p < 0.05$, when compared with TAM-treated animals (group II).

0.05, as compared to STZ-diabetic rats. The HDL-C elicited a significant increase by +37%, $p < 0.05$, as compared to STZ-diabetic rats. The intake of JFBE to STZ-diabetic rats produced a significant decline in serum TC, LDL-C, VLDL-C, TG, and LDL/HDL ratio levels by -14, -17, -31, -31, and -25%, respectively, $p < 0.05$; however, serum HDL-C level showed a significant increase of +11%, $p < 0.05$, as compared to STZ-diabetic rats (Table 3).

### DISCUSSION

During the last 2 decades, traditional medicines have become a topic of interest[26]. The present study was carried out to investigate hypoglycemic and hypolipidemic effects of JFEs in STZ-diabetic rats, compared to the reference hypoglycemic drug GLB. Five different JFEs were obtained and evaluated for their antioxidative activity *in vitro* using two different models: the DPPH$^{\cdot}$ free radical scavenging assay and the $Fe^{++}$chelating activity assay. The results revealed that the best two fractions that exhibited pronounced *in vitro* antioxidant activity were JFEE and JFBE and, in turn, both were chosen for subsequent *in vivo* experiments. This antioxidant activity may be attributed to the high phenolic content[14,27], as evident from the results obtained from the spectrometric analysis that showed the existence of isoquercitrin flavonoid in JFBE.

The experimental animal model used in this study was type II diabetes mellitus since a low single dose of STZ can destroy half of pancreatic β cells[28]. The mechanism by which STZ brings about its diabetic state includes pancreatic β-cell destruction, which make cells less active[6]. The significant high levels of serum FBG and %HbA1C in STZ-induced diabetic rats were lowered after the oral intake of either JFEE or JFBE. These changes were associated with a significant elevation of serum insulin level. The order of the hypoglycemic activity was JFEE > JFBE > GLB. The present results do not distinguish whether the effects observed are mediated at the level of alterations in fuel metabolism within the intestinal lumen *via* α-amylase inhibitory effects, β-cell function including regulation of insulin synthesis and secretion, or insulin action at target tissues[16,29,30]. Oral administration of JFEE or JFBE attenuated hyperglycemia and subsequently %HbA1C was declined due to the powerful hypoglycemic role of JFEE and JFBE[31]. The significant reduction of serum proteins was observed in STZ-diabetic rats. This decrease might be due to microproteinuria, which is an important clinical marker of diabetic nephropathy, and/or might be due to increased protein catabolism[32,33]. The serum protein content was returned to near normalcy after oral intake of either JFEE or JFBE to STZ-diabetic rats.

Lipid peroxidation is a marker of cellular oxidative damage initiated by reactive oxygen species[34]. It was reported that diabetics are highly sensitive to oxidative stress[35]. In STZ-diabetic animals, STZ generates nitric oxide, which is a powerful free radical oxidant[36] resulting in an increase in the serum level of lipid peroxides as noticed in this study due to cellular oxidation[37]. Production of lipid peroxides was significantly declined in JFEE > JFBE > GLB-treated STZ-diabetic rats. This may be explained on the basis of the presence of quercitrin flavonoid (resembles isoquercitrin present in JFE) that could attenuate the diabetic state by decreasing oxidative stress and preserving pancreatic β-cell integrity[38].

Abnormalities in lipid profile are common complications found in 40% of diabetics[39]. In this study, the STZ-diabetic rats elicited a significant increase in the serum TC, TG, LDL-C, and VLDL-C levels. High levels of TC and, more importantly, LDL-C in blood are major coronary risk factors[40]. The elevation of the serum lipid profile in diabetes is mainly due to an increase in free fatty acid mobilization from peripheral fat deposits, since insulin inhibits the hormone-sensitive lipase. Insulin deficiency may be responsible for dyslipidemia because insulin has an inhibitory action on HMG-CoA reductase, which results in increased production of LDL-C particle[41]. The administration of JFEE or JFBE to STZ-diabetic rats significantly increased serum HDL-C and decreased serum levels of TC, TG, VLDL-C, LDL-C, and LDL/HDL ratio. These effects were more pronounced in JFEE than in the case of JFBE. It was reported that there is a positive correlation between the risk of developing heart disease and raised TC and LDL-C concentrations, and negative correlation with HDL-C[42]. Thus, both JFEE and JFBE may have the ability to reduce the risk of cardiovascular disease.

### CONCLUSIONS

This present study investigated the potential hypoglycemic and hypolipidemic activities of both JFEE and JFBE compared to the hypoglycemic reference drug GLB. This point highlights the highly promising potential for substituting standard oral hypoglycemics in the treatment of type II diabetes. Studies are in progress to identify the active principle(s) in JFEs as well as to elucidate the underlying mechanism(s) of action.

## References

1. WHO Traditional Medicine Strategy 2002–2005. *WHO Publications*, 2002; 1–6.
2. Laakso, M. (2001) Insulin resistance and its impact on the approach to therapy of type 2 diabetes. *Int. J. Clin. Pract. Suppl.* **(121),** 8–12.
3. Matsui, T., Tanaka, T., Tamura, S., Toshima, A., Miyata, Y., and Tanaka, K. (2007) Alpha-glucosidase inhibitory profile of catechins and theaflavins. *J. Agric. Food Chem.* **55**, 99–105.
4. Nabel, E.G. (2003) Cardiovascular disease. *N. Engl. J. Med.* **349**, 60–72.
5. Grover, J., Yadav, S., and Vats, V. (2002) Medicinal plants of India with anti-diabetic potential. *J. Ethnopharmacol.* **81**, 81–100.
6. Marles, R. and Farnsworth, N. (1995) Antidiabetic plants and their active constituents. *Phytomedicine* **2**, 137–189.
7. Rahman, M., Nahar, N., Jabbar, M., and Mosihuzzaman, M. (1999) Variation of carbohydrate composition of two forms of fruit from jack tree (*Artocarpus heterophyllus* L.) with maturity and climatic conditions. *Food Chem.* **65**, 91–97.
8. Lin, C.N., Lu, C.M., and Huang, P.L. (2000) Flavonoids from *Artocarpus heterophyllus*. *Phytochemistry* **39(6),** 1447–1451.
9. Wei, B.L., Weng, J.R., Chiu, P.H., Hung, C.F., Wang, J.P., and Lin, C.N. (2005) Anti-inflammatory flavonoids from *Artocarpus heterophyllus* and *Artocarpus communis*. *J. Agric. Food Chem.* **53(10),** 3867–3871.
10. Mukherjee, B. (1993) *Traditional Medicine*. Mohan Primalani for Oxford and IBH Publishing, New Delhi. pp. 45–55.

11. Pua, C., Hamid, S., Rusul, G., and AbdRahman, R. (2007) Production of drum-dried jack fruit (*Artocarpus heterophyllus*) powder with different concentration of soy lecithin and gum Arabic. *J. Food Eng.* **78**, 630–636.
12. Fernando, M., Wickramasinghe, N., and Thabrew, M. (1991) Effect of *Artocarpus heterophyllus* and *Asteracanthus longifolia* on glucose tolerance in normal human subjects and in maturity-onset diabetic mellitus patients. *J. Ethnopharmacol*. **31(3),** 277–282.
13. Chandrika, U., Fernando, S., Wickramasinghe, N., and Wedage, W. (2002) Effects of proanthocyanidin and flavonoid fractions from hot water extract of Jack leaves (*Artocarpus heterophyllus*) on the blood glucose levels in healthy male Wistar rats. *Chem. Sri Lanka* **19(2),** 10.
14. Chandrika, U., Wedage, W., Wickramasinghe, N., and Fernando, S. (2006) Hypoglycemic action of the flavonoid fraction of *Artocarpus heterophyllus* leaf. *Afr. J. Trad. CAM* **3(2),** 42–50.
15. Shizuo, T. and Yoshiaki, S**.** (2006) Inhibitory effect of prenylated flavonoid in *Euchresta japonica* and *Artocarpus heterophyllus* on lipid peroxidation by interaction of hemoglobin and hydrogen peroxide. *Pharm. Biol*. **44(3),** 271–273.
16. Kotowaroo, M., Mahomoodally, M., Gurib-Fakim, A., and Subratty, H. (2006) Screening of traditional antidiabetic medicinal plants of mauritius for possible *α*-amylase inhibitory effects *in vitro*. *Phytother. Res*. **20(3)**, 228–231.
17. Khan**,** M., Omoloso, A., and Kihara, M. (2003) Antibacterial activity of *Artocarpus heterophyllus*. *Fitoterapia* **74(5),** 501–505.
18. Burits, M. and Bucar, F. (2000) Antioxidant activity of *Nigella sativa* essential oil. *Phytother. Res.* **14**, 323–328.
19. Gülçin, I. (2006) Antioxidant activity of caffeic acid (3,4-dihydroxycinnamic acid). *Toxicology* **217**, 213–220.
20. Akanksha, A., Srivastava, A., and Maurya, R. (2010) Antihyperglycemic activity of compounds isolated from Indian medicinal plants. *Ind. J. Exp. Biol*. **48**, 294–298.
21. Pari, L. and Umamaheswari, J. (2000) Antihyperglycemic activity of *Musa sapientum* flowers: effect on lipid peroxidation in alloxan diabetic rats. *Phytother. Res.* **14**, 136–138.
22. Chinta, G., Venkateswarulu, M., Prashanthi, K., Sujata, D., Pushpa, B., and Ranganayakulu, D. (2010) Anti-hyperlipidemic activity of the aqueous extract of the *Artocarpus heterophyllus* leaves in triton WR-1339 induced hyperlipidemic rats. *Drug Invent. Today* **2(1),** 25–28.
23. Bergmeyer, H.U., Ed. (1985) *Methods of Enzymatic Analysis*. 3rd ed. Vol. VIII. VCH Publishing. pp.154–160.
24. Ohkawa, H., Ohishi, N., and Yagi, K. (1997) Assay for lipid peroxides in animal tissues by thiobarbituric acid reaction. *Anal. Biochem.* **95**, 351–358.
25. Akiyama, T., Washino, T., Yamada, T., Koda, T., and Maitani, T. (2000) Constituents of enzymatically modified isoquercitrin and enzymatically modified rutin (extract). *J. Food Hyg. Soc. Japan* **41**, 54–60.
26. Ojewole, J. (2002) Hypoglycemic effect of *Clausena anisata* (Will) Hook methanolic root extract in rats. *J. Ethnopharmacol.* **81**, 231–237.
27. Soong, Y.-Y. and Barlow, P.-J. (2004) Antioxidant activity and phenolic content of selected fruit seeds. *Food Chem.* **88**, 411–417.
28. Eliza, A., Daisya, P., Ignacimuthub, S., and Duraipandiyan, V. (2009) Normoglycemic and hypolipidemic effect of costunolide isolated from *Costus speciosus* (Koen ex. Retz.) Sm. in streptozotocin-induced diabetic rats. *Chem. Biol. Interact.* **179**, 329–334.
29. Hara, Y. and Honda, M. (1990) The inhibition of *α*-amylase by tea polypphenols. *Agric. Biol. Chem.* **54(8),** 1939–1945.
30. Bansal, R., Ahmad, N., and Kidwai, J. (1981) Effect of oral administration of *Eugenia jambolana* seeds and chlororopamide on blood glucose level and pancreatic cathapsin B in rats. *Indian J. Biochem. Biophys.* **18**, 377–381.
31. Allen, D. (1964) *Hemoglobin within the Red Cell*, Academic Press, NewYork.
32. Bakris, G. (1997) Diabetic nephropathy. What you need to know to preserve kidney function. *Postgrad. Med.* **93**, 89.
33. Almdal, J. and Vilstrup, H. (1998) Strict insulin therapy normalizes organ nitrogen contents and the capacity of urea nitrogen synthesis in experimental diabetes in rats. *Diabetologia* **31**, 114–118.
34. Farber, J., Kyle, M., and Coleman, J. (1990) Biology of disease: mechanisms of cell injury by activated oxygen species. *Lab. Invest.* **62**, 670–679.
35. Baynes, J. (1991) Role of oxidative stress in development of complications in diabetes. *Diabetes* **40**, 405–412.
36. Kwon, N., Lee, S., Choi, C., Kho, T., and Lee, H. (1994) Nitric oxide generation from streptozotocin. *FASEB J.* **8**, 529–533.
37. Wakame, K. (1999) Protective effects of active hexose correlated compound (AHCC) on the onset of diabetes induced by streptozotocin in the rat. *Biomed. Res.* **20**, 145–152.
38. Coskun, O., Kanter, M., Korkmaz, A., and Oter, S. (2005) Quercetin, a flavonoid antioxidant, prevents and protects streptozotocin-induced oxidative stress and β cell damage in rat pancreas. *Pharmacol. Res.* **51**, 117–123.
39. Goldstein, D., Little, R., Lorenz, R., Malone, J., Nathan, D., Peterson, C., and Sacks, D. (2004) Test of glycemia in diabetes. *Diabetes Care* **27**, 1761–1773.
40. Soltani, N., Keshavarz, M., and Dehpour, A. (2007) Effect of oral magnesium sulfate administration on blood pressure and lipid profile in streptozotocin diabetic rats. *Eur. J. Pharmacol.* **560**, 201–205.

41. Murali, B., Upadhyaya, U., and Goyal, R. (2002) Effect of chronic treatment with *Enicostemma litorale* in non-insulin dependent diabetic rats. *J. Ethnopharmacol.* **81**, 199–204.
42. Jeppesen, J., Hein, H., Suadicani, P., and Gyntelberg, F. (1997) Relation of high TG- low HDL cholesterol and LDL cholesterol to the incidence of ischemic heart disease: an 8-year follow-up in the Copenhagen male study. *Arterioscler. Thromb. Vasc. Biol.* **17**, 1114–1120.
